# Anti-Inflammatory, Antioxidant, and Antimicrobial Potential of Natural Products

**DOI:** 10.3390/nu18172903

**Published:** 2026-09-04

**Authors:** Alex Graça Contato, Carlos Adam Conte-Junior

**Affiliations:** 1 Department of Agricultural, Livestock and Environmental Biotechnology, Faculty of Agricultural and Veterinary Sciences (FCAV), Sao Paulo State University (UNESP), Jaboticabal 14884-900, SP, Brazil; 2 Analytical and Molecular Laboratorial Center (CLAn), Institute of Chemistry (IQ), Federal University of Rio de Janeiro (UFRJ), Cidade Universitária, Rio de Janeiro 21941-909, RJ, Brazil; conte@iq.ufrj.br; 3 Center for Food Analysis (NAL), Technological Development Support Laboratory (LADETEC), Federal University of Rio de Janeiro (UFRJ), Cidade Universitária, Rio de Janeiro 21941-598, RJ, Brazil; 4 Laboratory of Advanced Analysis in Biochemistry and Molecular Biology (LAABBM), Department of Biochemistry, Federal University of Rio de Janeiro (UFRJ), Cidade Universitária, Rio de Janeiro 21941-909, RJ, Brazil; 5 Graduate Program in Biochemistry (PPGBq), Institute of Chemistry (IQ), Federal University of Rio de Janeiro (UFRJ), Cidade Universitária, Rio de Janeiro 21941-909, RJ, Brazil; 6 Graduate Program in Food Science (PPGCAL), Institute of Chemistry (IQ), Federal University of Rio de Janeiro (UFRJ), Cidade Universitária, Rio de Janeiro 21941-909, RJ, Brazil; 7 Graduate Program in Veterinary Hygiene (PPGHV), Faculty of Veterinary Medicine, Fluminense Federal University (UFF), Niterói 24220-000, RJ, Brazil; 8 Graduate Program in Chemistry (PGQu), Institute of Chemistry (IQ), Federal University of Rio de Janeiro (UFRJ), Cidade Universitária, Rio de Janeiro 21941-909, RJ, Brazil

Natural products have historically represented an important source of bioactive molecules with applications in nutrition, health promotion, and disease management [1,2]. Plants, fungi, microorganisms, and other natural sources contain diverse classes of secondary metabolites and complex bioactive matrices capable of interacting with multiple molecular and cellular targets [3,4,5]. In recent years, growing evidence has demonstrated that these compounds may modulate inflammatory signaling, oxidative stress, immune responses, apoptosis, microbial communities, and tissue homeostasis, thereby attracting increasing interest in potential functional food ingredients, nutraceuticals, and complementary therapeutic agents [6,7]. The scope of this Special Issue was therefore centered on understanding the biological activities and mechanisms of natural products, particularly their capacity to modulate inflammation, oxidative damage, and microbial activity, as well as their potential applications in the prevention and management of disease.

Inflammation and oxidative stress are closely interconnected biological processes involved in the initiation and progression of numerous chronic and acute diseases [8,9]. Although inflammation is an essential component of host defense and tissue repair, persistent or excessive inflammatory responses may contribute to tissue injury and disease progression [10]. Similarly, an imbalance between reactive oxygen species (ROS) production and antioxidant defenses may amplify inflammatory signaling, impair cellular function, and promote pathological changes [11]. Consequently, natural products capable of simultaneously targeting oxidative and inflammatory pathways have received considerable attention as potential strategies for maintaining physiological homeostasis.

Several contributions to this Special Issue address the potential of natural compounds to attenuate inflammation and tissue injury in experimental models of disease. Sun et al. investigated the effects of betulin, a lupane-type pentacyclic triterpenoid, in a mouse model of 5-fluorouracil-induced intestinal mucositis. Betulin treatment alleviated body weight loss, diarrhea, reduced food intake, and histological intestinal damage, while the 200 mg/kg treatment was associated with reduced inflammatory and apoptosis-related markers, including *Tnf*, *Nos2*, Bax, and caspase-3, together with increased Bcl-2 expression. The authors also observed treatment-associated changes in gut microbial diversity and composition, although these microbiota findings were considered exploratory because of the limited sample size [Contribution 1].

Inflammatory skin disorders represent another important area in which natural products may provide complementary therapeutic opportunities. Han et al. evaluated a chemically characterized cannabidiol-dominant *Cannabis sativa* L. inflorescence extract in a mouse model of atopic dermatitis. The extract reduced mast cell infiltration and serum IgE levels, modulated Th2-associated cytokines, and inhibited MAPK, NLRP3 inflammasome, and JAK1/STAT6 signaling. In addition, treatment increased the expression of skin barrier proteins, including filaggrin and involucrin, and improved skin hydration. These findings suggest that standardized *C. sativa* preparations may exert multi-target effects relevant to chronic inflammatory skin conditions [Contribution 2].

The liver is another organ highly susceptible to the consequences of metabolic dysfunction, oxidative imbalance, and inflammation. Kim et al. investigated an Rb1-enriched red ginseng saponin fraction in a mouse model of metabolic dysfunction-associated alcoholic liver disease. The treatment reduced hepatic steatosis and inflammatory lesions and, importantly, was associated with suppression of lipopolysaccharide-induced TLR4 internalization. The authors proposed that regulation of TLR4 trafficking and inhibition of LPS–TLR4 interactions contributed to the suppression of M1 macrophage polarization and the hepatoprotective effects observed in the experimental model [contribution 3].

The interaction between natural products, intestinal inflammation, and the gut microbiota is also prominently represented in this collection. Guo et al. investigated total flavonoids from sea buckthorn leaves in a dextran sulfate sodium-induced colitis model. The flavonoid fraction improved colonic damage and intestinal barrier integrity through the modulation of ZO-1, E-cadherin, and MUC2, while reducing TNF-α and IL-6 levels. Furthermore, treatment altered the gut microbial community, including an increase in *Akkermansia*, and promoted short-chain fatty acid production. These findings highlight the potential of plant-derived flavonoids to act through interconnected microbiota, metabolite, epithelial barrier, and immune pathways, while also demonstrating the potential value of sea buckthorn leaves as a source of bioactive compounds [Contribution 4].

The relationship between natural products and the gut microbiota extends beyond intestinal disorders and may have implications for distant organs and systemic health. Araújo-Rodrigues et al. investigated digestive fractions obtained from mushroom biomass of *Trametes versicolor*, *Hericium erinaceus*, and *Pleurotus ostreatus*, together with gut microbiota-derived metabolites such as short-chain fatty acids and γ-aminobutyric acid. In human microglial cells, mushroom digestive fractions and short-chain fatty acids reduced reactive oxygen species production, whereas mushroom fractions, butyric acid, and GABA improved behavioral outcomes in transgenic *Caenorhabditis elegans* models associated with Tau hyperphosphorylation and β-amyloid toxicity. The study provides evidence supporting the potential contribution of mushrooms and microbiota-derived metabolites to the modulation of oxidative stress and neurodegeneration-associated processes [Contribution 5].

Oxidative stress and inflammation are likewise central to respiratory disorders. Umsumarng et al. evaluated an anthocyanin-rich fraction obtained from Kum Akha black rice in cellular and animal models of NLRP3 inflammasome-driven lung inflammation. The fraction contained substantial amounts of cyanidin-3-*O*-glucoside and peonidin-3-*O*-glucoside and displayed antioxidant activity. In LPS- and ATP-stimulated A549 cells, treatment suppressed NLRP3-associated inflammatory responses and reduced IL-6, IL-1β, and IL-18 expression and secretion. In mice, the fraction attenuates inflammatory cytokines and histopathological lung lesions. These results highlight the potential of pigmented rice and its anthocyanin-rich fractions as functional food resources capable of modulating inflammasome-associated inflammation [Contribution 6].

Beyond isolated phytochemicals, fermentation represents an important strategy for modifying natural matrices and potentially generating compounds with enhanced biological activity. Ma et al. investigated an extract of *Atractylodes japonica* rhizome fermented with the plant-derived lactic acid bacterium *Lacticaseibacillus paracasei* IJH-SONE68 in a mouse model of wheat gliadin-induced food allergy. The fermented extract reduced the anaphylaxis score and modulated serum immunological parameters, including total IgE, IFN-γ, and IL-4. The authors suggested that fermentation may alter the bioactive properties of medicinal herbal extracts and contribute to the modulation of Th1/Th2 responses, supporting the potential of microbial fermentation as a strategy for developing functional preparations from traditional medicinal resources [Contribution 7].

A related approach was explored by Sumneang et al., who investigated fermented *Houttuynia cordata* juice in a rat model of lipopolysaccharide-induced sepsis. Pretreatment with the fermented juice prevented cardiac dysfunction and attenuated inflammatory, oxidative, and apoptotic responses. These effects were associated with changes in IL-1β, TLR4, NF-κB, malondialdehyde, cleaved caspase-3, and Bax/Bcl-2-related markers, together with reductions in LDH and CK-MB, indicating decreased cardiac injury. This contribution reinforces the potential of fermented plant-derived preparations to modulate multiple pathological pathways simultaneously and illustrates the relevance of fermentation-derived products to cardiovascular protection under inflammatory conditions [Contribution 8].

Fermented foods themselves constitute an important component of the relationship between natural products, microorganisms, and human health. Black et al. reviewed the effects of kefir consumption on the human oral and gut microbiome. Their review emphasizes the potential of this fermented beverage to influence microbial ecosystems and, consequently, systemic health. However, the authors also point out that the available evidence from high-quality human clinical trials remains limited. Further controlled clinical investigations are therefore necessary to establish the safety, efficacy, and clinical relevance of kefir consumption in relation to oral and intestinal microbiota and metabolic health [Contribution 9].

Mushrooms represent another particularly relevant source of natural compounds with diverse biological activities. In their narrative review, Contato and Conte-Junior examined the nutritional and therapeutic potential of *Hericium erinaceus*, with particular emphasis on its antioxidant, anti-inflammatory, antimicrobial, and neuroprotective properties. The authors discuss the contribution of polysaccharides, terpenoids, including hericenones and erinacines, and phenolic compounds to the biological activity of this mushroom. The potential stimulation of nerve growth factor synthesis further supports interest in *H. erinaceus* in the context of neurodegenerative diseases. At the same time, the review emphasizes the importance of standardized extraction procedures, pharmacokinetic studies, and large-scale clinical trials to establish the translational potential of this mushroom as a functional food, nutraceutical, or complementary therapeutic agent [Contribution 10].

The Special Issue also encompasses broader perspectives on the mechanisms through which natural compounds may influence chronic diseases. Messeha et al. reviewed the role of Nrf2 activation as a potential therapeutic target for flavonoids in aging-related osteoporosis. Aging is associated with increased oxidative stress and disruption of antioxidant defenses, while dysregulation of the Nrf2/Keap1 pathway may contribute to an imbalance between bone formation and resorption. The review discusses the potential of flavonoids to counteract oxidative stress and exert anti-osteoporotic effects, positioning Nrf2 modulation as a promising mechanism linking natural compounds, redox homeostasis, and bone health [Contribution 11].

Finally, Ussia et al. provided a systematic review of the effects of virgin and extra virgin olive oil consumption on cardiovascular health and disease prevention. Following the PRISMA statement and PICO framework, the authors identified 17 clinical studies and reported evidence associating virgin olive oil consumption, including extra virgin olive oil, with reduced cardiovascular risk and improvements in cardiometabolic biomarkers. Attention was given to the polyphenols present in extra virgin olive oil and their potential contribution to the cardioprotective effects of the Mediterranean dietary pattern. Nevertheless, the authors emphasized the need for larger multicenter clinical studies to further clarify the preventive effects of olive oil consumption on cardiovascular outcomes [Contribution 12].

Taken together, the contributions included in this Special Issue demonstrate the remarkable diversity of natural products and the multiple biological pathways through which they may influence human health. The studies encompass purified compounds, plant extracts, fermented preparations, mushrooms, pigmented cereals, fermented foods, and dietary products, illustrating that the biological potential of natural products cannot be restricted to a single chemical class or mechanism of action. Instead, their effects frequently involve interconnected processes, including oxidative stress, inflammatory signaling, immune regulation, apoptosis, intestinal barrier integrity, gut microbiota modulation, and tissue-specific responses.

An important feature of this collection is the diversity of experimental approaches represented. The published studies include cellular models, animal models, simulated gastrointestinal digestion, microbiota-related investigations, narrative reviews, and systematic reviews. This diversity provides complementary evidence regarding both mechanistic pathways and the broader relevance of natural products to nutrition and human health. At the same time, several contributions emphasize the limitations that must be addressed before promising findings can be translated into clinical or nutritional applications, particularly regarding standardization, bioavailability, pharmacokinetics, dose optimization, safety, and the need for adequately designed clinical trials.

Through this Special Issue, we aimed to bring together recent advances illustrating how natural products can contribute to the modulation of inflammation, oxidative stress, microbial communities, and disease-related pathways. The twelve (12) contributions collected here cover a broad spectrum of natural sources and health outcomes, ranging from intestinal, hepatic, and dermatological disorders to neurodegeneration, respiratory inflammation, cardiovascular dysfunction, osteoporosis, and food allergy. Collectively, these studies reinforce the importance of multidisciplinary approaches combining nutrition, natural product chemistry, microbiology, pharmacology, molecular biology, and translational research.

We hope that this collection will stimulate further investigations into the mechanisms underlying the biological activities of natural products and encourage the development of well-characterized, standardized, and scientifically validated functional ingredients and complementary therapeutic strategies. Editing this Special Issue has been a valuable opportunity to bring together diverse perspectives on the potential of natural products and to contribute to the advancement of research at the interface of nutrition, bioactive compounds, and human health.
